# Apoferritin is maintaining the native conformation of citrate synthase *in vitro*

**Published:** 2018-12-31

**Authors:** Yuri V Sergeev, Monika B Dolinska, J Fielding Hejtmancik

**Affiliations:** Ophthalmic Genetics and Visual Function Branch, National Eye Institute, USA

**Keywords:** apoferritin, ferritin, chaperoning-like property, protection of protein catalytic activity, molecular crowding

## Abstract

Ferritin, a member of a family of iron storage proteins, is expressed in conditions of oxidative or thermal stress in the cell. Ferritin widely found in human tissues including the eye and brain. Increased expression under oxidative or temperature stress conditions and protective effect on cell viability suggest that apo form of ferritin (apoferritin) may have a role in the formation or maintenance of the native conformation of proteins. To test this hypothesis, we studied the influence of apoferritin on the unfolding and refolding of citrate synthase (CS) *in vitro.* Here we show that at stoichiometric amounts apoferritin is remarkably protecting the CS catalytic activity, stabilize the aggregation of CS under heat stress and act as chaperone-like molecules in these folding reactions *in vitro*. Furthermore, apoferritin promote the functional refolding of CS after guanidinium hydrochloride denaturation. Finally, these results confirm that apoferritin has chaperone-like activity *in vitro* and suggests that apoferritin might have a role in protection and maintaining of protein native conformation.

## Introduction

Ferritin, the major iron storage protein, is found in a wide variety of different human tissues, especially spleen and liver but also including the eye and brain, and is a member of family of iron storage proteins. Ferritin consists of a mixture of variable amounts of two species, the light chain (FTL) with 175 amino acids and a molecular weight of 19,000 and the heavy chain (FTH) with 183 amino acids and a molecular weight of 21,000. Iron-free ferritin molecules (apoferritin) form hollow spheres composed of 24 protein subunits with major motif consisting of a 4-helix bundle.^1–3^ This molecule can store up to 4500 iron atoms in ferric hydroxide phosphate complexes.^2^ Ferritin is a translationally regulated heat shock protein of avian reticulocytes as previously been demonstrated.^2^ Recent data on ferritin gene transcription^4,5^ emphasizes that it is oxygen stress that is the toxic agent; ferritin in fact, is protecting cells from oxidant damage. Ferritin has been shown to increase in response to stress and inflammation and sequester iron-II in the cell. Apoferritin has been shown to exist in significant amounts in various tissues, and perhaps even to be compartmentalized within tissues. Ferritin consists of a mixture of a complex of apoferritin-iron hydroxide and about 25% ‘free apoferritin’. Free apoferritin is the natural apoferritin^6,7^ and also present in human serum.^6,8^ The ferritin L-chain does not have the ability to inhibit Fenton mediated hydroxyl radical production as does H-chain ferritin.^9^ However ferritin L-chain is playing an important role in protection against oxidative damage, based on the similar responses of the L-chain DNA promoter and the promoters of antioxidant response proteins, such as QR and TRR, all which have ARE sequence.^10^ The protective role of apoferritin has also been demonstrated in cell culture where addition of apoferritin protects human leukemia or endothelial cells from oxidative stress^11,12^ and in a mouse multiple sclerosis model, in which apoferritin treatment limited the tissue damage.^13^ While these effects might be mediated in part through changes in iron metabolism, they do confirm a role for apoferritin in defense against oxidative and other stress.

Increased expression under oxidative or temperature stress conditions and protective effect on cell viability at these conditions suggest that apoferritin might have a role in the formation or maintenance of the native conformation of proteins. Because of potential partners of apoferritin during the stress at present are not well defined, we tested the chaperone-like properties of these proteins with using CS, a well characterized protein used by others to study refolding and mechanism of chaperone action *in vitro*.^14^ Here we show that apoferritin suppresses *in vitro* thermal aggregation of CS in a highly efficient manner, preserve the enzymatic activity of this protein under thermal stress and act as effective chaperone in folding reactions.

## Materials and methods

### Proteins

Proteins used in this work were ferritin and thyroglobulin (Tg) (GE Healthcare, Piscataway, NJ), aldolase, apoferritin and ferritin (Sigma-Aldrich Inc, St. Louis, MO), α-crystallin (Stressgene Biotechnologies, Victoria, British Columbia, Canada) and citrate synthase (Roche Applied Science, Indianapolis, IN).

### Aggregation assay

Proteins were dialyzed overnight in Buffer A: 100mM Tris HCl, pH 8.0 at 37°C, followed by centrifugation for 30 min at 8,000x g at 4°C to remove protein aggregates. Protein concentration was determined using A260/280 or the Bradford method.^15^ Kinetics of protein aggregation were determined by measuring of apparent absorption due to light scattering at 360nm (A360) in a DU-650 spectrophotometer (Beckman, USA) equipped with a Peltier temperature controller. Substrates and chaperone-like proteins were mixed in 400μL black walled cuvettes at molar ratios as described in the text. The scattering in each cell was recorded automatically each 1 min over a 1 hour interval at fixed temperature (37°C and 43°C). For analysis of heat treated precipitates, samples were incubated at 43°C for 15 min, centrifuged at 10Kg × 30 min, the precipitates were dissolved in 4% SDS and subjected to SDS-PAGE and Western blot analysis using a polyclonal rabbit antiserum to CS purchased from Nordic Immunological Laboratories (Tiburg, Netherlands) and anti-horse spleen ferritin antibody developed in rabbit (Sigma-Aldrich, Saint Louis, USA).

### Enzymatic CS activity

Enzymatic activity of CS was assayed using oxaloacetate and acetyl-CoA as substrates as described by Srere et al.^16^ Briefly, a reaction cocktail was prepared by mixing 7.5ml of Buffer A, 2ml of 100mM L-malic acid, 1ml of 50mM nicotinamide adenine dinucleotide, 2ml of 2mM acetyl coenzyme A sodium salt in Buffer A, 1ml of 560 units/ml malic dehydrogenase enzyme solution in Buffer A and 15.5ml of deionized water. Aliquots of the reaction cocktail were preincubated in teflon-stopped cuvettes in a Peltier chamber at 37°C until the A340nm reading was constant. Then 0.4units/ml of intact CS in the absence or the presence of apoferritin, Tg or α-crystallin mixed with 1ml of reaction cocktail and the increase in A340nm was recorded for 5 min. The same procedure was used for the heat-inactivated CS samples obtained by different incubations at 43°C (0, 5, 10, 20, 30, 45 and 60 min). All reagents used were from Sigma-Aldrich (St. Louis, MO).

### Thermal protection during the heat denaturation

Thermal denaturation was measured at 360 using the DU-6500 spectrophotometer (Beckman Coulter, Fullerton, CA). Aldolase or CS loaded to make final concentration of 1.5μM in the 400μL teflon-stopped cuvettes containing *Buffer B*: 50mM Na-phosphate, pH7.0, 1mM EDTA, 1mM DTT. Chaperone-like proteins were added in equimolar concentration. Temperature was changed from 15°C to 90°C with a rate 1°C/min. Percent of protein protection is calculated using the following expression *100x* (*A360*_*prot*_*−A360*_*prot+chap*_)*/A360*_*prot*_^*max*^*, %*, where A360 is the reading of the UV-spectrophotometer at 360nm for the protein (*prot*) and protein in the presence of chaperone-like molecule at molecular ratio 1:1 (*prot+chap*).

### CS reactivation

Samples of chaperone-like proteins and controls were transferred in the *Buffer A* and were frozen at −70°C in 200μL aliquots. Initial concentrations for frozen proteins (100x) were chosen to make the final concentration of 5:1 molar ratio of the chaperone-like protein to the CS and allowing following100-fold dilution in refolding buffer. Thawed CS samples were denatured in the *Buffer C*: 6M GdmCl, 0.1M Tris-HCl, pH8.0, 20mM DTT for 1.5hr and were used in refolding experiment. Refolding was initiated by diluting the denatured CS 100-fold into refolding *Buffer D*: 0.1M Tris-HCl, pH 8.0, 2mM EDTA, 0.15M NaCl. The CS refolding in the presence of chaperones or controls was performed in same conditions maintaining the molar ratio 1:5 of CS to chaperone. Samples of denatured CS and the 5-fold excess of chaperone were loaded simultaneously. CS activity was assayed like that of described above. Light scattering was measured using Cary Eclipse fluorometer (Varian Inc., Palo Alto, CA) equipped with 4 Peltier-controlled 4ml cuvettes allowing to carry out four independent measurements in same time at 25°C. Samples were thoroughly stirred in each cuvette during the experiment. Both the excitation and emission wavelengths were 500nm. Spectral band was 2.5nm for both excitation and emission.

### Apoferritin structure and electrostatic calculations

The structure of apoferritin molecule was taken from the Brookhaven Protein Database (PDB: 1aew, http://www.rcsb.org/pdb). Accessible areas of exposed and inner surfaces were calculated by the Surface Racer program version 3.0. Electrostatic potential was determined by solving a Poisson-Boltzman equation using focusing method realized in the program DelPhi. The complete atomic structure including hydrogen atoms was used as a model of the ferritin protein shell. The following below calculations were repeated for ferritin without iron and loaded with iron as described in the text. In first model the apoferritin structure is surrounded by and the interior protein cavity of 30Å radius filled by aqueous solvent described by the Debye-Huckel formula. In the second model, the iron-containing core of ferritin was placed in protein cavity. The iron core was generated using 643 non-overlapping copies of the modified fragment of mixed-valent polyiron oxo complex structure^16^ from the Cambridge Database of Chemical Structures (file YALKAX10). The solvent and protein have dielectric constants of 80 and 4, respectively. This model included a cubic grid of 129×129×129 nodes. An initial calculation was carried out for the matrix occupying of 48.5% of nodes with a 2Å distance between grid points. The resulting grid energies were used to determine the electrostatic energies on a grid of the same size with a 1Å step and with the matrix occupying 96% of the nodes. The resulting electrostatic potential mapped to the protein surface using the program UCSF Chimera (http://www.cgl.ucsf.edu/chimera).

## Results

### Apoferritin and Tg suppress the CS thermal aggregation

**Figure 1:**
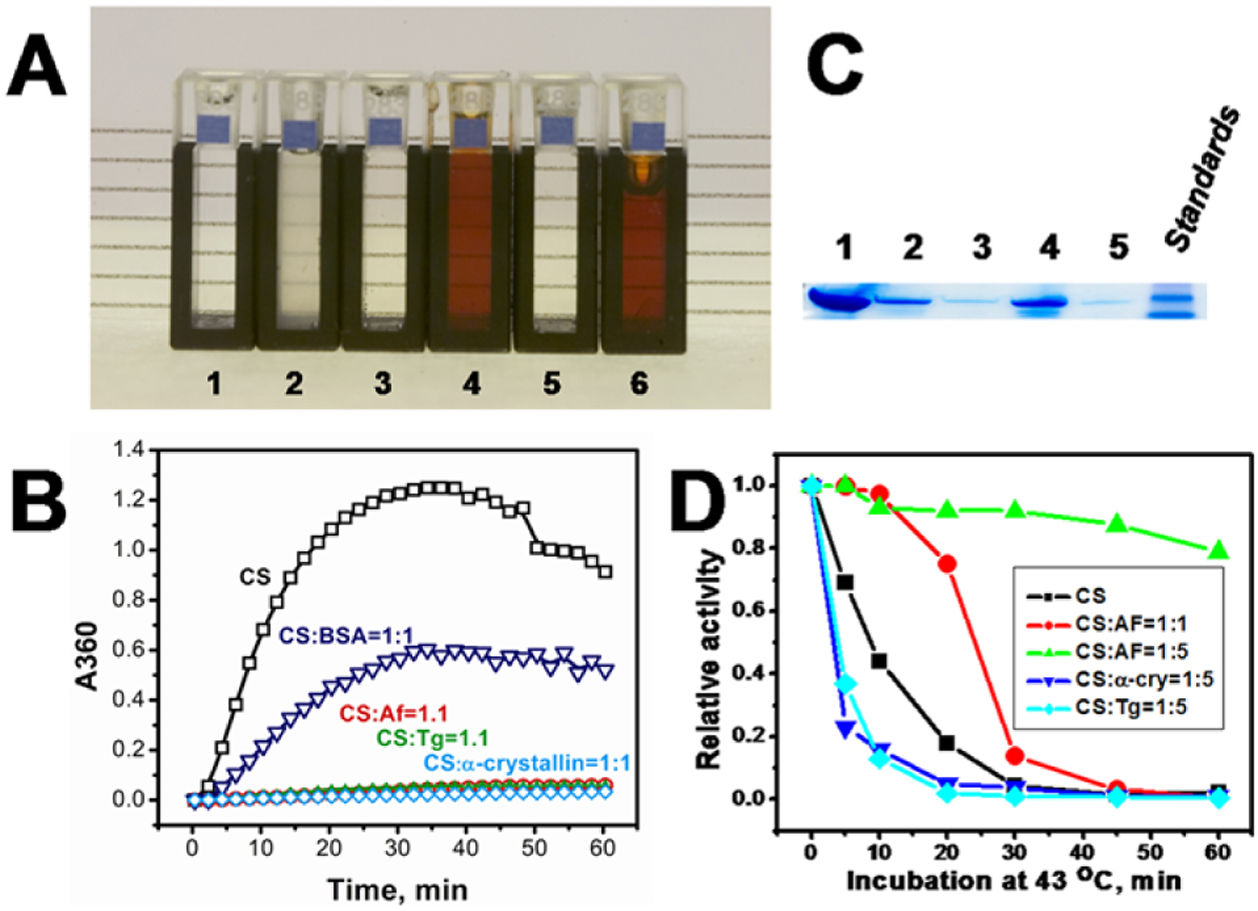
Apoferritin suppress CS aggregation and preserves CS activity.

The effect of thermally induced CS aggregation by ferritins is shown in Figure 1**. Panel A** show cells containing Buffer A (1), CS (1.1μM) in the absence (2) or presence (3) of apoferritin, ferritin (4) or Tg (5) incubated for 15 min at 45°C with a 1:1 molar ratio of protein to CS, and ferritin by itself (6). The relative absorption at 360nm for CS (7.5μM) in the absence (□) or presence of apoferritin (○), Tg (◻), α-crystallin (◊) and BSA (◻), all in molecular ratios of 1:1 is shown in **Panel B**. **Panel C** represent PAGE of heat treated precipitates in lanes 1–6: CS control (1), CS incubated at 43°C for 15 min (2), in the presence of a 1:1 molar ratio of apoferritin (3), ferritin (4), Tg (5); and standards (6). The enzymatic activity of heat inactivated CS in the absence (■) or in the presence of apoferitin in molecular ratios 1:1 (red ●) and 1:5 (green ▲), respectively, and in presence of α-crystallin (blue ▼) and Tg (light blue ◻), both at a 1:5 molecular ratio of CS to chaperone-like protein is shown on Panel D.

### Apoferritin protects an enzymatic activities of citrate synthase and aldolase

**Figure 2:**
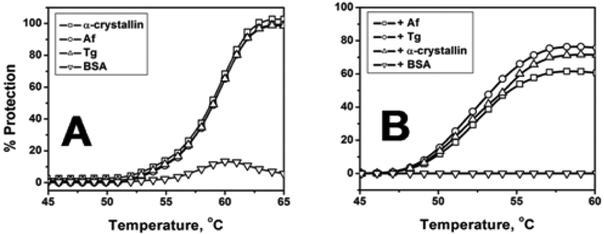
CS and aldolase are protected against heat-induced protein denaturation by apoferritin.

In contrast to bovine serum albumin (BSA), chaperone-like molecules, α-crystallin, apoferritin (Af), thyroglobulin (Tg), protect aldolase (Figure 2A) and CS (Figure 2B) under heat-induced protein denaturation. The light scattering was registered at 360nm. All measurements obtained using the temperature-controlled Beckman DU-650 spectrophotometer. The molar ratios were 1:1 of aldolase or CS to each of chaperone-like molecule. Protection % calculated as described in Methods section.

### Apoferritin increase yield of the CS refolding

**Figure 3:**
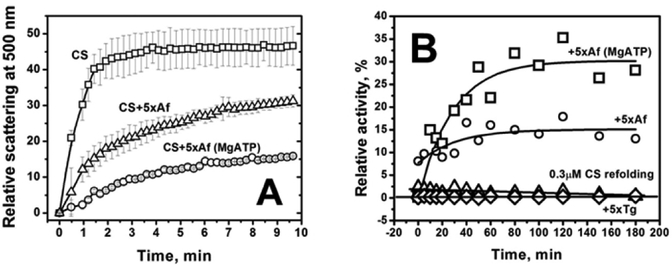
CS aggregation suppression by apoferritin was determined by light scattering at 500nm and reactivation of CS during the time course of the CS refolding.

CS was completely denatured in the Buffer C and 100-fold diluted in the Buffer D at 25°C. Figure 3A shows light scattering at 500nm in the time course of CS refolding of the 0.75μM CS in Na-phosphate buffer in the absence or in the presence of 5-fold excess of apoferritin is shown. Light scattering curves and error bars are determined by averaging of 4 simultaneous measurements obtained for 4 independent protein samples. Kinetics of the CS reactivation in the absence (open triangles) or in the presence (open circles) of 5-fold excess of apoferritin in the Buffer D is shown in Figure 3B, respectively. Reactivation of CS in the presence of Tg is shown by open rhombs. Curves of the best fit are shown by solid lines. Measurements of scattering or activity in the Na-phosphate buffer performed in the presence of 10mM MgCl_2_ and 2mM ATP are labeled as MgATP.

### Electrostatic potential of the ferritin surface

**Figure 4:**
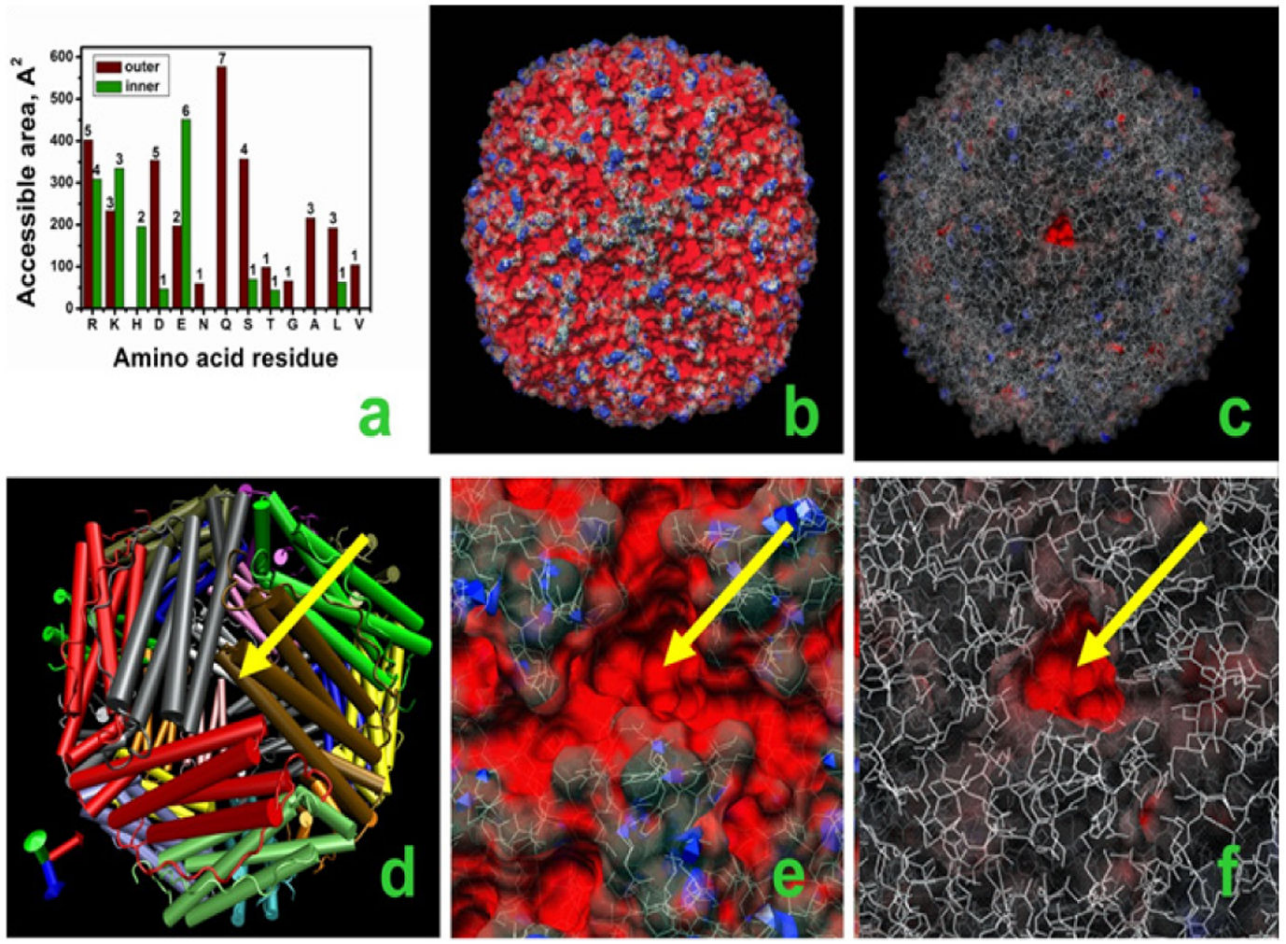
Modeling effects of the iron-containing core in the chaperone function of ferritin.

Cumulative accessible areas of residues with accessibility above the cutoff of 35Å^2^ are shown in **Panel 4a** by brown and green bars for outer and inner residues, respectively. The number of residues of each amino acid at the surface of the apoferritin monomer is shown as the number above of each bar. The molecular surface of apoferritin colored with regards to the electrostatic potential is shown in **Panel 4b** from the side of 4-fold channel. Values of the electrostatic potential are shown in **Panels 4b**, **4e** and **4f** by red (< −25kT/e), gray (between −25kT/e and 25kT/e) and blue (>25kT/e) colors. **Panel 4c** demonstrate the structure of a modified fragment of mixed-valent polyiron oxo complex^16^ used to generate the ferrihydrite core of ferritin. Oxygen and iron atoms are colored blue and brown, respectively. **Panel 4d** represent the apoferritin structure with each subunit colored differently. Helices are shown as cylinders. Similar views of the electrostatic potentials of apoferritin **(Panel 4e)** and iron-loaded ferritin **(Panel 4f)** mapped to the molecular surface. Yellow arrows on **Panels 4d–4e** show the location of the 3-fold channel (Figure 4).

## Discussion

Apoferritin is a potential multidrug transporter with a wide-range applications in nanomedicine.^17^ Here we demonstrated that apoferritin suppresses *in vitro* thermal aggregation of citrate synthase in a highly efficient manner, preserve the enzymatic activity of this protein under thermal stress and act as effective chaperone in folding reactions. This is also compatible with the appearance of the homologous but aferrous protein artemin from brine shrimp *Artemia franciscana* embryos contemporaneously with remarkable physiological stress resistance.^18^ Artemin as well as apoferritin suppresses heat-induced aggregation of CS i*n vitro* and artemin protect cell from thermal stress.^19^ Analysis of light scattering curves in this work suggested that CS is protected from thermal stress in the presence of iron-containing ferritin. According to our data, the presence of ferritin in solution does not protect the solubility of citrate synthase due to thermal stress as confirmed by the SDS-PAGE of insoluble protein (Figure 1C).

Figure 1 shows samples of CS after incubation for 15 min at 43°C alone, or in the presence of apoferritin, ferritin or used as control in our experiments Tg. Cell 2, containing CS alone, and cell 4 containing CS and ferritin show increased turbidity, indicating protein aggregation, although this is somewhat obscured by the reddish discoloration seen in cell 4 due to the presence of iron oxide. In contrast cells 3 and 5, containing CS with apoferritin and Tg respectively, maintained levels of transparency like cell 1, containing only buffer B. This suggests that apoferritin and Tg have protein aggregation suppression activities similar to those of α-crystallin, another large oligomeric protein with well characterized chaperone function.^20^ The effects of apoferritin and Tg on light scattering by CS subjected to thermal stress are shown further in Figure 1B. Incubation of CS alone at 43°C gives increasing light scattering to an A360 of about 1.2 over the first 30 minutes. While addition of an equimolar concentration of BSA, a protein also demonstrated some chaperone-like properties amount of apoferritin or Tg dramatically suppresses light scattering by CS by about 96% at 30 min, with little other change over 1 hour, results similar to those seen with α-crystallin. Confirmation of these findings is shown in Figure 1C, an SDS-acrylamide gel of proteins incubated for 15 min at 43°C as in Figure 1A. The samples are then centrifuged at 10K x g, 4°C for 30 min to pellet the insoluble faction, which was then dissolved in 4% SDS. A control CS sample is shown in lane 1 for comparison. Incubation of CS in the absence of any other protein (lane 2) or in the presence of ferritin (lane 4) results in considerably more aggregated protein than incubation in the presence of equimolar amounts of apoferritin or Tg (lanes 3 and 5). Thus, in contrast to iron containing ferritin, both apoferritin and Tg have chaperone-like properties and can protect CS from thermal aggregation.

While Tg and α-crystallin only maintain the clarity of CS solutions, apoferritin could protect the enzymatic activity of CS during incubation at 43°C. After heating CS for 30 minutes at 43°C the enzymatic activity falls almost 10-fold, and a 5-fold excess of α-crystallin or Tg speeds the loss of activity (Figure 1D). In contrast, most CS activity is preserved for the first 10 minutes of incubation at 43°C in the presence of equimolar amounts of apoferritin, and about 80% of CS enzymatic activity is maintained for 1 hour in the presence of a 5-fold molar excess of apoferritin (Figure 1D).

The remarkable role of apoferritin and Tg in protein aggregation suppression (Figure 1A & Figure 1B) suggests that chaperone-like proteins might protect CS against of thermal denaturation. Calculations based on apparent scattering data obtained at 360nm for the CS denaturation in Na-phosphate buffer show that apoferritin, Tg and α-crystallin are protecting CS against of aggregation in less degree demonstrating the 61%, 76% and 71% of protection at same temperature respectively (Figure 2B). To demonstrate that chaperone-like action of apoferritin and Tg is not specific to protein tested we also shown the protective effect on aldolase. The effect of chaperone-like proteins demonstrated on Figure 2A. All three chaperone-like proteins α-crystallin, thyroglobulin and apoferritin show close to 98% aldolase aggregation protection at 64°C. The addition BSA to aldolase or to CS show no or low protection at that temperature. This suggests that apoferritin as well Tg or α-crystallin, help to resist the heat-induced thermal denaturation *in vitro*.

With a purpose to understand the role of apoferritin in refolding of CS the reactivation experiments were performed. CS was completely denatured in the Buffer C and 100-fold diluted in the phosphate buffer at 25°C. Light scattering data obtained for the 0.75μM CS in the time course of refolding (Figure 3A) suggest that protein aggregation dominates folding reaction.^14^ In the presence of 5-fold excess of apoferritin CS show decrease of scattering suggesting that apoferritin suppresses the protein aggregation during CS refolding. Results of CS reactivation in the presence of apoferritin are demonstrated in Figure 3B. Incubation of 0.3μM CS at 25°C in the absence of any chaperone-like protein as well as in the presence of Tg or in the presence of BSA (data not shown) demonstrated very low CS reactivation (<3%). However, in the presence of apoferritin the CS activity yield is changed significantly and even more in the presence of MgATP in the buffer (Figure 3B). A role of MgATP in this process still not well understood. However, the presence of apoferritin suppresses the CS scattering and might have a role in the maintaining of native CS during CS refolding *in vitro*.

To estimate the fraction of charged residues exposed on the surface of the protein, cumulative accessible surface areas (Figure 4A) for each amino acid residue located on the inner or outer surface of apoferritin (PDB: 1aew) were calculated. The majority of residues (79%) exposed on the outer surface are polar (32% or 802.4Ǻ^2^) or polar charged (47% or 1187Ǻ^2^) in contrast to those located on the inner surface, 95.6% of which are polar (7.9% or 114.3Ǻ^2^) or polar charged (87.7% or 1263Ǻ^2^). Thus, most residues exposed at the apoferritin surface are polar or polar charged. To understand the possible role of these residues and the effect of the iron-containing core in defining the surface properties of apoferritin, the electrostatic potential of the surface of iron-free (filled with solvent) and iron-loaded ferritins were mapped as shown in Figure 4B & Figure 4C, respectively. The structure of iron core was modeled as follows. X-ray and electron diffraction studies suggest that Fe3+ is deposited in the ferritin core as the hydrous ferric oxide mineral ferryhydrite or a similar structure.^2,21,22^ While the crystallographic structure of the ferritin core is uncertain, the data suggest a mixed-valent polyiron oxo complex^18^ made up of double hexagonally closest packed oxygen atoms in a stacked lattice, with a majority of Fe3^+^ being octahedrally coordinated.^22^ This basic structure was used to model the iron core in the protein cavity and to calculate an electrostatic potential around ferritin loaded with iron. Differences in the surface electrostatic potentials of iron-free and iron-loaded ferritin near the 3-fold channel are shown in Figure 4E & Figure 4F. The basic structure of apoferritin is shown in Figure 4D for orientation. Most of the apoferritin surface has a negative potential, shown by a red color in Figure 4E. In contrast, when an iron core is present in the molecule the surface has a more neutral potential as demonstrated by the gray color seen in Figure 4F, although a small area in the center, corresponding to the 3-fold channel felt to be the likely route of iron entry into the protein shell,^1,3^ still has a significant negative potential that reflects the continuous availability of the channel for iron entry. Thus, the presence of the iron core in ferritin appears to modulate the surface electrostatic potential. The proposed electrostatic mechanism for apoferritin is different from that of recently published,^22,23^ which suggests that thiols or the inter-subunit disulphide bond might play a role in the homologous artemin chaperone function.

The effect of excluded volume^24^ might play a role in protein aggregation suppression and in protection against the heat-induced thermal denaturation. However, on our opinion, this effect has less role in protection and maintaining native protein conformation, because of in our reactivation experiments thyroglobulin failed to demonstrate either the protection or the maintaining of the CS catalytic activity (Figure 1D & Figure 3B).

Protein–chaperone interaction is transient by nature and influenced by rotational and translational diffusion, the transition state barrier for the transient complex formation, short-range hydrophobic and long-range electrostatic intermolecular forces acting between the proteins. In our opinion, all these forces and the apoferritin reaction surface might play a role in the protection and maintaining of the native CS fold. Our experiments designed to elucidate the role of these forces and the reaction surface in the mechanism of chaperone-like function of apoferritin is underway.

### Limitations of our study

We have demonstrated that large protein molecule such as apoferritin might play a role in the protection of catalytic activity of other proteins and therefore could be useful for nanomedical applications. Currently our conclusions are limited to the *in vitro* study and should be further investigated in a future.

## Conclusion

Apoferritin suppresses *in vitro* thermal aggregation of CS in a highly efficient manner, preserve the enzymatic activity of citrate synthase under thermal stress and act as effective chaperone in folding reactions. These results confirm that apoferritin has chaperone-like activity *in vitro* and suggests that apoferritin might have a role in protection and maintaining of protein native conformation.
